# Polidocanol versus hypertonic glucose for sclerotherapy treatment of reticular veins of the lower limbs: study protocol for a randomized controlled trial

**DOI:** 10.1186/1745-6215-15-497

**Published:** 2014-12-19

**Authors:** Matheus Bertanha, Marcone Lima Sobreira, Carlos Eduardo Pinheiro Lúcio Filho, Jamil Victor de Oliveira Mariúba, Rafael Elias Farres Pimenta, Rodrigo Gibin Jaldin, Andrei Moroz, Regina Moura, Hamilton Almeida Rollo, Winston Bonetti Yoshida

**Affiliations:** Department of Surgery and Orthopedics, School of Medicine, São Paulo State University (UNESP), Rubião Junior s/n, CEP 18.618-970 Botucatu, SP Brazil; Department of Bioprocess and Biotechnology, School of Pharmaceutical Sciences, São Paulo State University (UNESP), Jaú Highway, KM01, ZIP CODE 14801-902 Araraquara, SP Brazil

**Keywords:** Glucose solution, Hypertonic, Sclerosing solutions, Sclerotherapy, Telangiectasis, Varicose veins, Veins

## Abstract

**Background:**

The prevalence of chronic venous disease is high and occurs more frequently in females. According to the clinical, etiological, anatomical, and pathological classification (CEAP) definition, the reticular veins are included in the C1 class and are mainly associated with aesthetic complaints. Several invasive techniques are used for treatment, including mini phlebectomy, laser ablation, and radiofrequency ablation. However, a wide range of sclerosing agents may serve as minimally invasive alternatives, promoting chemical sclerosis of the vein wall. Although this technique is routinely performed around the world, there is no consensus on the most efficacious and safe chemical agent to be used.

**Methods/design:**

Inclusion criteria are women between 18 and 69 years old with at least 10 cm long reticular veins in the lower limbs, on the outer side of the leg/thigh. Patients with CEAP 2 to 6, or with allergies, pregnancy, performing breastfeeding, or with any dermatologic or clinical problems will be excluded. Patients with venous ultrasound mapping showing involvement of saphenous trunks and/or a deep venous system will also be excluded. Patients will be randomized into two groups, one receiving 75% pure glucose and the other group receiving 0.2% polidocanol diluted in 70% glucose. Just one limb and one session per patient will be performed. The sclerosing agent volume will not exceed 5 mL. Clinical follow-up will include visits on days 7 and 60, always with photographic documentation.

**Discussion:**

This project aims to enroll 96 patients and subject them to a double-blind treatment after the randomization process. The design is intended to evaluate efficacy through a primary end point and safety through a secondary end point. Forty-eight patients have currently been enrolled. Preliminary results for these patients showed that 25 received treatment, 2 were excluded, and 22 returned after 7 days and showed no greater adverse events. To date, establishing efficacy criteria has not been possible, and no patients have reached the 60-day return point. These data may help doctors choose the best chemical agent for the treatment of reticular veins.

**Trial registration:**

ClinicalTrials.gov Identifier: NCT02054325, 3/02/2014.

**Electronic supplementary material:**

The online version of this article (doi:10.1186/1745-6215-15-497) contains supplementary material, which is available to authorized users.

## Background

According to the definition of the clinical, etiological, anatomical, and pathological classification (CEAP), the reticular veins are included in the C1 class and are mainly associated with aesthetic complaints. Several invasive techniques are commonly used for treatment, including mini phlebectomy, laser ablation, and radiofrequency ablation. However, a wide range of sclerosing agents may serve as minimally invasive alternatives, promoting chemical sclerosis of the vein wall. Reticular veins have a diameter no greater than 3 mm and are straight, bluish, and located in the subcutaneous tissue of the lower limbs [1]. They have the same complex etiology of all types of primary venous insufficiency such as hormonal issues, family history, and standing position [2, 3]. However, despite its common occurrence, the condition remains poorly understood [4]. Sclerotherapy is the preferred treatment for some patients because of its simplicity and reproducibility [5, 6], and consists of an intraluminal sclerosing agent application to trigger endothelial injury, with subsequent luminal occlusion of these segments. Many agents, such as hypertonic saline and hypertonic glucose as hyperosmolar sclerosing agents, as well as ethamolin® and polidocanol as detergent agents [5, 7], are known to be effective. Hypertonic osmotic solutions cause dehydration and destruction of endothelial cells. Hypertonic dextrose (or glucose) and sodium tetradecyl sulfate are commonly used due to their low cost and safety [8–10]. Conversely, polidocanol acts as a detergent on vein wall lipids, destroying the cellular cement and causing endothelial maceration; this process effectively occludes the vein [11–14]. Due to its efficacy and manipulability, the use of polidocanol is quite common throughout the world. Usually, it is mixed with a wide variety of diluents, such as distilled water, air, or hypertonic glucose. However, some collateral damage can occur from these mixtures, including necrosis, hyperpigmentation stains, allergic reactions, and systemic disorders. Chest pain, cough, anaphylaxis, scotoma, and gas embolization are unusual, but serious adverse events have already been reported in other studies due to this agent, although these issues are commonly related to miscalculated dosages [15–17].

Chemical sclerotherapy is slightly painful and the discomfort is limited to the puncture site, which warrants a free anesthesia procedure. The procedure is usually well tolerated by patients and has a rapid recovery period. However, the use of sclerotherapy for reticular veins is controversial because of its association with blemishes and other risks. However, evidence of these risks is equivocal [2]. Recent studies have shown that various formations of polidocanol are effective in the performance of sclerotherapy for reticular veins [11]. Results from our study have shown positive effects in the treatment of reticular veins using a 0.2% polidocanol solution and 70% hypertonic glucose. Additionally, there was a low occurrence of stains and no major collateral effects. The Food and Drug Administration approved polidocanol as a medicinal sclerosing agent in the United States in 2010, and was soon followed by several European countries. The formulation of polidocanol described above is available for sclerotherapy in Brazil despite the lack of appropriate scientific literature on this treatment. Therefore, the aim of this study is to assess the benefits and safety of a reticular vein treatment using a formulation of 0.2% polidocanol with 70% hypertonic glucose in Brazilian patients.

### Hypothesis

We hypothesize that 0.2% polidocanol plus 70% hypertonic glucose is superior to 75% hypertonic glucose alone in promoting the disappearance of reticular veins of the lower limbs. Moreover, we hypothesize that 0.2% polidocanol plus 70% hypertonic glucose is not inferior to 75% hypertonic glucose in terms of safety.

## Methods and design

### Trial design

This study is a single-center, prospective, randomized, triple-blind trial to compare the safety and efficacy of two sclerosing agents (0.2% polidocanol plus 70% hypertonic glucose versus 75% hypertonic glucose) in women over 60 days.

### Patient enrollment and randomization

A convenience sample of patients undergoing treatment for mild varicose veins will be employed. Patient information will be entered into an electronic database, and participants will later be contacted to attend an outpatient evaluation. In the first evaluation, patients who have a reticular vein measuring at least 10 cm long in the outer-side of the leg and/or thigh will be invited to join the study. The sample size was estimated to be 96 treated limbs (see “Statistical analyses”). All subjects will be informed of study risks and will be required to sign an informed consent form approved by the institutional review board. The random allocation schedule will be generated using the computer program Stat Trek (http://stattrek.com/Tables/Random.aspx).

Two groups will be established for the treatment of reticular veins, including either 0.2% polidocanol diluted in 70% glucose or 75% glucose alone. A trained nurse will store all data and remain in charge of arrangements, such as preparing the solutions used in the treatments, throughout the duration of the study. Solutions will be prepared in a specific room (different from the office) using sterile 10 mL syringes. Syringes will be identified according to a random number given to each patient.

### Ethics

This trial is being conducted in accordance with the principles of Helsinki’s Declaration, ISO14155 and Good Clinical Practices guidelines. The Research Ethics Committee of the Botucatu Medical School, from the Paulista State University, São Paulo State, Brazil, approved this trial, which was registered under protocol number CEP4127-2012. A written informed consent was obtained from all subjects before enrollment. Subjects can withdraw from this trial whenever at their own discretion, with no prejudice. The *ClinicalTrials.gov* identifier for this trial is NCT02054325 and was obtained on February 3, 2014.

### Clinical and pre-treatment measures

Patients will be included if they present one or more reticular veins with a minimal length of 10 cm, and no edema or palpable tortuosity. A duplex ultrasonography will then be performed prior to treatment. Clinical data, such as comorbidities, demographic features, and other relevant information, will be collected for later analysis. All excluded patients will be referred for other clinical follow-up techniques at the institution.

### Inclusion and exclusion criteria

#### Inclusion criteria

Women, having one or more reticular veins measuring at least 10 cm long on the outer side of the leg and/or thigh with a clinical classification of C1 CEAP [18] (which means absence of varicose veins), aged between 18 and 69 years, who agree to the terms of the research, sign the free consent form, available to make the required appointments necessary for follow-up, consent to avoid pregnancy for at least three months after the treatment, and do not use anticoagulant drugs (Additional file 1).

#### Exclusion criteria

Males, females with CEAP 2 to 6 [18], low mobility, peripheral arterial disease, known allergy history to the drugs used in this study (glucose and polidocanol), presence of dermatitis in the lower limbs and presence of comorbidities (such as diabetes mellitus, heart failure, respiratory failure, uncontrolled hypertension, hypothyroidism or hyperthyroidism), pregnancy, breastfeeding, pulmonary hypertension, prior deep venous thrombosis (DVT), familiar history of DVT, known hypercoagulable states or thrombophilia, asthma, migraine, or anyone that does not agree with any of the research terms (Additional file 1).

### Treatment area

To define the treatment area, on the outer side of the lower limbs (thigh and leg) and using the fibular head projection as an anatomical reference point, a longitudinal imaginary line measuring 25 cm (up) and 15 cm (down) will be drawn using the fibular head as a starting point. Two additional lines will be drawn parallel to the first one, with one marked at 7.5 cm anterior and the other at 7.5 cm posterior, which define the limited area for treatment (600 cm^2^; Additional file 2). In this area, one or two reticular veins, measuring from 10 cm to 20 cm in total length, will be treated with a minimum of 10, and a maximum of 30, punctures and with 5 mL as the maximum volume of medication over the treatment duration. Each puncture will not exceed 0.3 mL of volume.

### Photographic registry

Photos of patients will be taken to register the early and late results of the treatment area (pre-treatment). A high definition camera (D7000 Nikon™ Lens: AF-S Nikkor™ 18–105 mm 1:3.5-5.6G) will be used to obtain these pictures. The target area, described above, will be photographed before treatment (pretreatment) to prevent a possible time bias related to the patient’s illness. Patients will be placed in a standing position on a platform with the target area facing the camera, which will be placed 1.0 m away. A high-definition standardized scale (attached to the platform) will be used to perform all measurements. The room will be arranged with ideal lighting.

Pictures will be stored in files for later analysis using ImageJ™ software. Each photo will be identified with code numbers (according to the randomized number of the protocol), matching the moment of the study (Day 0 (D0) and D60). ImageJ software will be used to measure the length of the treated veins by turning pixels into centimeters using a 2 cm line drawn upon the standardized scale. Measurements will be taken from the photos before and after the study.

### Treatment (D0)

After the photographs are taken, the two groups will be treated similarly to eliminate all the reticular veins in the area of the selected lower limb. All procedures will be performed by the same physician. A rigorous conventional sclerotherapy technique will be followed, consisting of careful injections to prevent leakage of fluid into the subcutaneous tissue. The procedure will be performed until veins become undetectable. The maximum volume allowed per puncture will not exceed 1 mL. A 3 mL luer-lok plastic syringe BD® will be used with a 13 × 4 Terumo® needle (27 G1/2). Bandages will be performed with a cotton ball (approximately 0.3 cm in diameter) attached to a square piece of plaster tape (measuring approximately 2 × 3 cm) right after the completion of the procedure to stop backflow. Before discharge, the target area will be submitted to elastic compression using a 1.5 m long elastic band (Atadress®) for one day [19].

All treatment data will be gathered, including the minimal volume of solution necessary for the whole disappearance of all the reticular veins in the target area, number of punctures, location of the target area, possible allergic reactions, and existence of minimum varicose veins in the target area. Additionally, a form will be given to the patient to fill out to report discomfort or pain triggered by the treatment (Additional file 3). Patients will be given careful oral and written instructions on how to proceed after treatment (Additional file 4). All patients will receive one tube of cream containing 0.5% sodium heparin to treat any initial blemishes, to be used twice per day for two weeks.

### Post-treatment (D7)

On approximately the seventh day, a new clinical evaluation will be performed (7 ± 2 days after the treatment session; D7). At this appointment, early results of the treatment will be evaluated, focusing on adverse events (minor or major) such as allergies, edema, respiratory complaints, syncope, scotoma, suspected DVT, and phlebitis. If identified, these adverse events will be recorded. Should phlebitis occur, it will be released with microneedle punctures (13 × 4.5 BD®) and gentle compression. An image from the target area will be recorded and placed at the same position as described above.

### Post-procedure (D60)

After 60 ± 15 days, the last clinical evaluation will be performed. Efficacy issues will be recorded and a new photo register (from the target area) will be performed, according to the procedures described above. In addition, any new adverse event that has been reported for the patient or noticed during the physical examination (allergies, edema, respiratory complaints, syncope, scotoma, suspected DVT, phlebitis, ulceration) will be registered. In case of phlebitis, treatment will be the same as described above.

### Outcomes

#### Primary endpoint – efficacy

Analysis of the primary endpoint (efficacy) will be based on the ability of each treatment to promote the disappearance of the reticular veins after 60 days of treatment (D60). Efficacy will be scored according to commonly used visual aesthetic aspects [2]. Photo analysis will be used to evaluate efficacy (from D0 to D60), which will be performed by two external physicians (both vascular surgeons) who are blinded to treatment modality. According to the image, these judges will assign scores for each patient as follows: 5 (excellent – complete healing at the target area); 4 (very good – presence of up to 1 cm of non-healed veins in length at the target area); 3 (good – presence of 1 cm to 3 cm of non-healed veins in length at the target area); 2 (fair – presence of 3 cm to 5 cm of non-healed veins in length at the target area), 1 (bad – presence of 5 cm to 9 cm of non-healed veins in length at the target area), and 0 (unsuccessful – presence of more than 9 cm of non-healed veins in length at the target area) (Additional file 5). Prior to these analyses, both judges will be trained with 10 random patients to standardize the methods of measurement.

If there are conflicting results in the scores assigned by the two judges (divergence in scores up to 1 point), a third previously trained judge will be assigned to independently score that image, and the final score will be the average of the scores from the three judges. However, if there are conflicting results in the scores assigned by the two judges (divergence in scores greater than 2 points), a conference among the two judges with a third previously trained judge will be arranged to define a consensus on the final score. All these procedures will be carried out in a blind manner.

#### Secondary endpoint – safety

Any type of adverse event (major or minor) that arises through the period of the study (D0 to D60) will be tracked. Reports, such as pain due to the procedure (discomfort reported by the patient, caused by needle punctures, and/or agent indwell), will be evaluated using the “Patient Satisfaction Questionnaire”. Adverse events that will be directly analyzed at the secondary endpoint (Additional file 3) are stains (limited to the target area), hematomas (limited to the target area), edema, and phlebitis (limited to the target limb). DVT is a severe, but unusual, complication with few existing reports documenting its effects [15]. However, this complication will also be recorded if it occurs. All of these data will be collected during medical visits (D0 to D60).

The photos taken at the end of the study (D60) will be used to evaluate irreversible skin changes such as hyperpigmentation. Two external physicians (both vascular surgeons), blinded to which modality of treatment was carried out, will score the changes as follows: 5 (excellent – no visible hyperpigmentation at the target area); 4 (very good – presence of only one point of hyperpigmentation up to 1 cm in length at the target area); 3 (good – presence of 2 to 3 points of hyperpigmentation up to 1 cm in length at the target area); 2 (fair – presence of 4 to 5 points of hyperpigmentation up to 1 cm in length, or one hyperchromatic line, up to 5 cm in length, at the target area); 1 (bad – presence of more than 5 points of hyperpigmentation up to 1 cm in length, or one hyperchromatic line, with more than 5 cm and less than 9 cm in length, at the target area); and 0 (unsuccessful – presence of a hyperchromatic line larger than 9 cm in length, or the total extension of the vein presenting hyperpigmentation, at the target area) (Additional file 6). Any conflicting results in the scores assigned by the two judges will be handled as described in the “Primary endpoint” section above.

Other data will be gathered in the analysis such as demographic data, skin color, and skin changes at the target area (pain, phlebitis, stains). Major adverse events, such as deep venous thrombosis, will also be reported throughout the study.

### Statistical analyses

Based on alpha = 0.05, and assuming a standard deviation in the primary endpoint score of 1.5, with 48 participants in each arm, we have >80% power to detect a minimal difference in scores of 0.9. Only one extremity will be included per patient. It is expected that 10% of patients will be lost during follow-up. To promote the most statistically rigorous method, we will use a larger sample size of at least 106 patients. Data will be evaluated either by Student’s *t*-test or non-parametric Mann–Whitney test (if relevant conditions are not met). Categorical variables (e.g., proportions) will be compared between groups using the χ^2^ or Fisher’s exact test when necessary. Scores between the groups will be compared with Mann–Whitney test. All statistical analyses will be performed using STATA® v11 (Stata Corp, College Station, TX, USA). All analyses will be performed on an intent-to-treat basis.

### Expected adverse effects

Both selected study drugs (75% hypertonic glucose and 0.2% polidocanol) are widely used as sclerosing agents in aesthetic venous procedures. There are a few reports of 75% hypertonic glucose usage resulting in major complications. However, polidocanol is associated with some reports of adverse events such as gas embolism and deep venous thrombosis. Pain, local edema, stains, phlebitis, and ulcers are minor adverse events related to these agents. If any other relevant effects occur, they will be reported to the Ethics Committee.

### Preliminary results

Forty-eight patients are currently enrolled; 25 patients have received treatment, 2 patients were excluded due to selection error, and 22 showed no major adverse effects during D7. However, the majority of patients had small local hematoma. Nevertheless, determining the efficacy of the sclerosing agent is currently not possible, given that no patient reached D60. We expect to answer some questions regarding the safety and efficacy of these sclerosing agents, including the advantages and limitations of each. Finally, we hope to determine the superiority of one agent over the other.

### Funding

This study has received financial support from “Sao Paulo Research Foundation – FAPESP”, under protocol number 2012/11034-0.

## Discussion

Several studies have shown sclerotherapy to be feasible in the treatments of reticular veins using various sclerosing agents [20–22]. However, none has investigated the effects of polidocanol diluted at 0.2% in 70% glucose when compared to 75% glucose, commonly used in these procedures. Our goal is to compare the safety and efficacy of these two agents, as well as to determine a possible superiority of one over the other. Secondary endpoints consist of evaluating pain triggered by the sclerosant agent, as well as the occurrence of adverse events like phlebitis and stains [15]. Additionally, we intend to report the occurrence of “difficult to treat” hyperchromic stains in patients submitted to this type of treatment. This information, analyzed judiciously, will help vascular surgeons and dermatologists choose the best therapeutic modality.

## Trial status

This trial has been actively recruiting patients since September 2013. The completion date for this study is estimated as December 2014.

## Electronic supplementary material

Additional file 1: **Inclusion and exclusion criteria of this trial.** (DOC 39 KB)

Additional file 2: **Representative picture of anatomic area of treatment.** (DOCX 68 KB)

Additional file 3: **Patient satisfaction questionnaire form.** (DOC 272 KB)

Additional file 4: **Instructions for the patient.** (DOC 29 KB)

Additional file 5: **Primary end point.** Score of disappearance of reticular veins treated. (DOC 34 KB)

Additional file 6: **Secondary end point.** Score of pigmentation. (DOC 32 KB)

## Abbreviations

CEAP: Classification of chronic venous disease
DVT: Deep venous thrombosis
D0: Pre-treatment period
D7: Seventh day
D60: Sixtieth day.
