# Supplementary material for: Polidocanol versus hypertonic glucose for sclerotherapy treatment of reticular veins of the lower limbs: study protocol for a randomized controlled trial
Source: Trials. 2014 Dec 19;15:497. doi: 10.1186/1745-6215-15-497 (PMC4301449; doi:10.1186/1745-6215-15-497)
Supplement: Supplementary file 1 — Additional file 1: Inclusion and exclusion criteria of this trial. (DOC 39 KB) [file 13063_2014_2369_MOESM1_ESM.doc]

Additional file 1

| **Inclusion criteria:** | **Exclusion criteria:** |
| --- | --- |
| Females | Male |
| Reticular vein longer than 10cm at the outer side of lower limb | Clinical classification of chronic venous disease different from C1 |
| Clinical classification of chronic venous disease - C1(mild venous disease) | Restricted mobility |
| Minimum age of 18 year-old | History of peripheral arterial disease |
| Maximum age 69 year-old | Dermatitis on the application site |
| Agreed and signed the free consent form | Known allergy to polidocanol or glucose |
| Availability to attend to appointments | History of Diabetes Mellitus |
| Warned about avoiding pregnancy in the periprocedural time (3 months) | Pregnancy |
| Free of anticoagulant drugs | Heart failure |
|  | Respiratory failure |
|  | Uncontrolled hypertension when under medication |
|  | Hypothyroidism |
|  | Hyperthyroidism |
|  | Pulmonary hypertension |
|  | Previous DVT |
|  | Family history of DVT |
|  | Hypercoagulable states or known thrombophilia |
|  | Asthma |
|  | Migraine |
|  | Didn’t agree with the research terms |
