# Supplementary figures and images for: Polidocanol versus hypertonic glucose for sclerotherapy treatment of reticular veins of the lower limbs: study protocol for a randomized controlled trial
Source: Trials. 2014 Dec 19;15:497. doi: 10.1186/1745-6215-15-497 (PMC4301449; doi:10.1186/1745-6215-15-497)

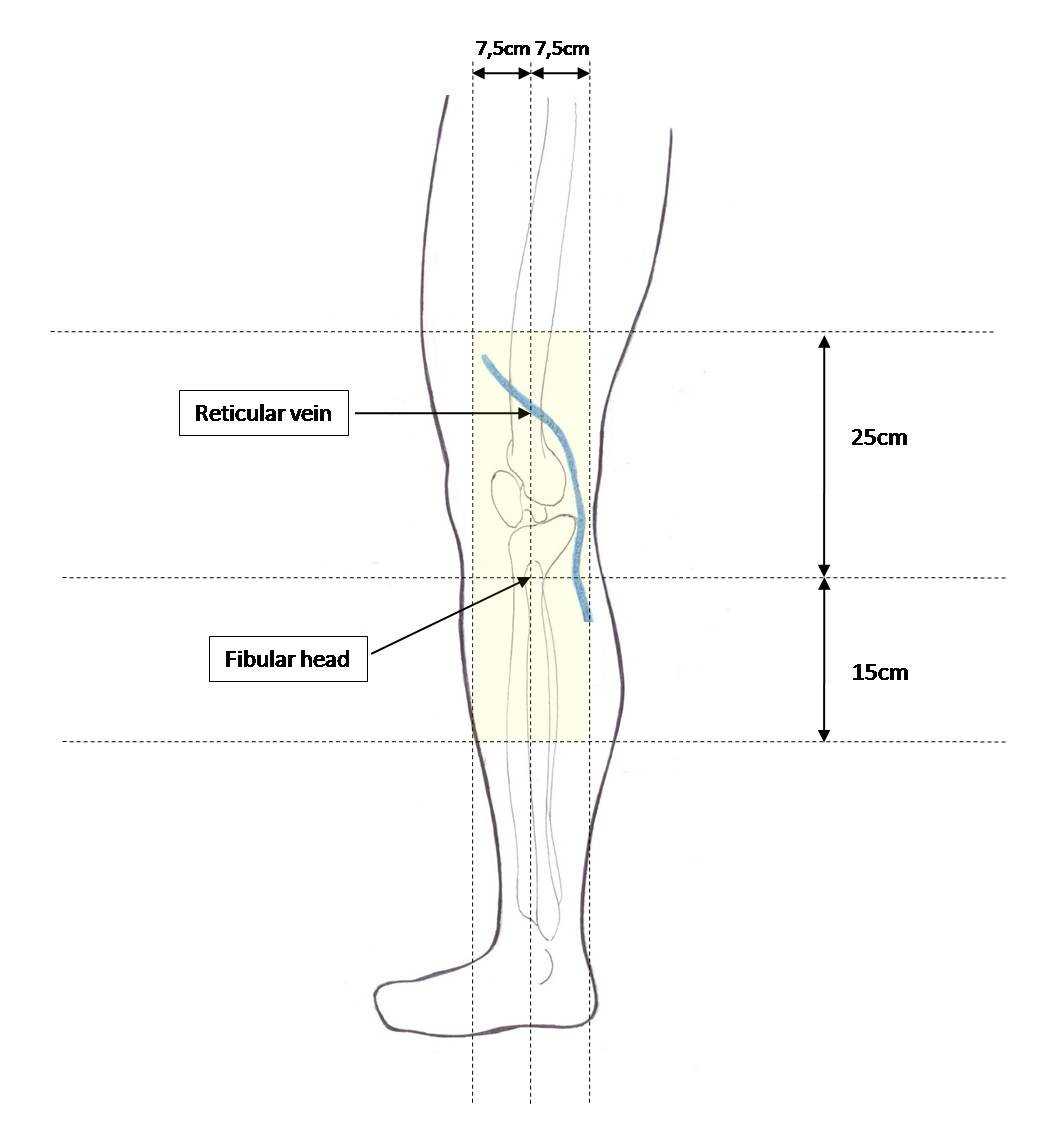

Supplement: Supplementary file 2 — Additional file 2: Representative picture of anatomic area of treatment. (DOCX 68 KB) [file 13063_2014_2369_MOESM2_ESM.docx]
