# Supplementary material for: Polidocanol versus hypertonic glucose for sclerotherapy treatment of reticular veins of the lower limbs: study protocol for a randomized controlled trial
Source: Trials. 2014 Dec 19;15:497. doi: 10.1186/1745-6215-15-497 (PMC4301449; doi:10.1186/1745-6215-15-497)
Supplement: Supplementary file 3 — Additional file 3: Patient satisfaction questionnaire form. (DOC 272 KB) [file 13063_2014_2369_MOESM3_ESM.doc]

PROTOCOL NUMBER ________

PATIENT SATISFACTION QUESTIONNAIRE

1) Which is the level of discomfort that the reticular veins in your lower limb causes in your life, such as for wearing certain clothing (skirts and shorts)?

( ) 0 = None

( ) 1 = Very few

( ) 2 = Few

( ) 3 = Moderate

( ) 4 = Great

( ) 5 = Extreme.

2) Score the pain you eventually felt during this treatment session: 0 is no pain and 10 is the worst pain you've ever felt in your life:

( ) 0 ( ) 1 ( ) 2 ( ) 3 ( ) 4 ( ) 5 ( ) 6 ( ) 7 ( ) 8 ( ) 9 ( ) 10


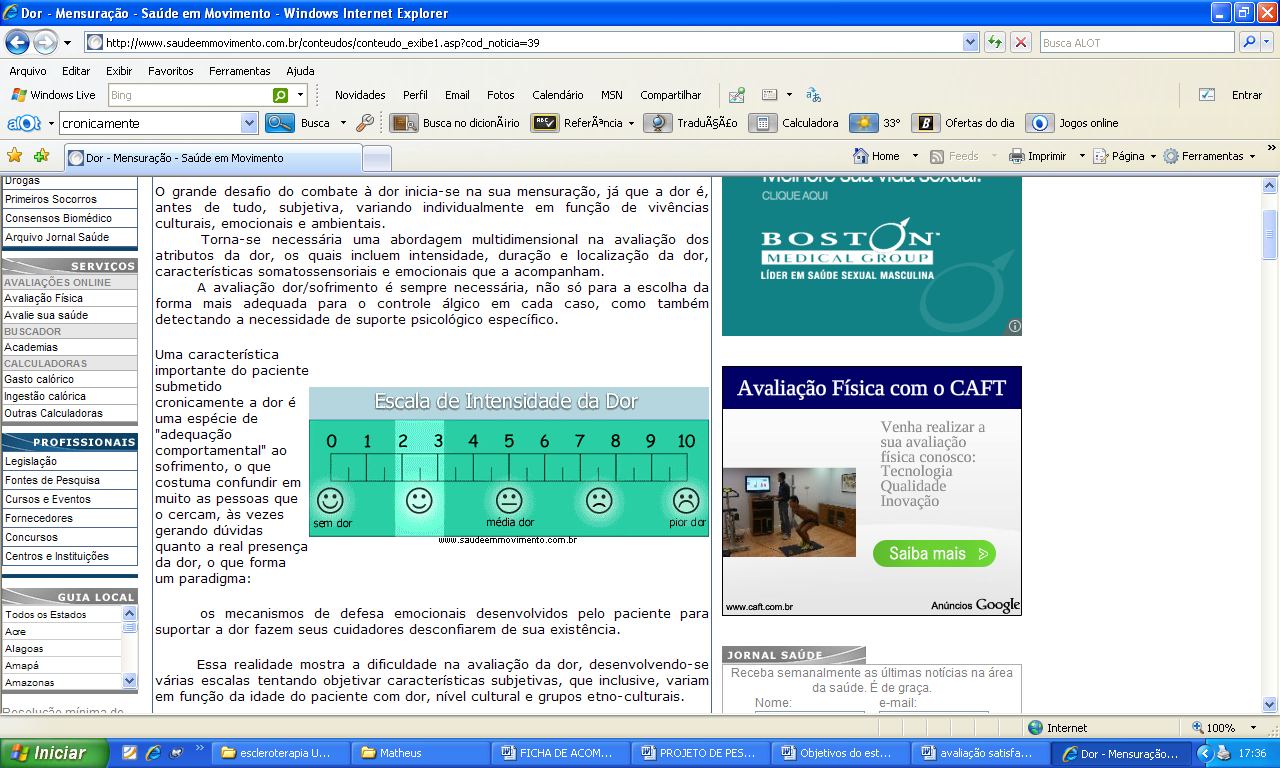


3) Compared to similar treatments that you’ve been submitted to previously, the procedure you were submitted to today caused:

( ) less pain ( ) similar pain ( ) more pain ( ) never done any treatment before

4) In your opinion, the worst part of the treatment was due to:

( ) Needle ( ) Liquid ( ) for both liquid and needle ( ) cannot explain

* Please DO NOT IDENTIFY YOURSELF; we will use only the protocol number. This is very important for this research.
