# Supplementary material for: Polidocanol versus hypertonic glucose for sclerotherapy treatment of reticular veins of the lower limbs: study protocol for a randomized controlled trial
Source: Trials. 2014 Dec 19;15:497. doi: 10.1186/1745-6215-15-497 (PMC4301449; doi:10.1186/1745-6215-15-497)
Supplement: Supplementary file 4 — Additional file 4: Instructions for the patient. (DOC 29 KB) [file 13063_2014_2369_MOESM4_ESM.doc]

To : ______________________________________________ Date : ______________

**INSTRUCTIONS FOR THE PATIENT**

1) Leave the bandage intact on your limb during the first day after the procedure, for a better result.

2) If swelling happens, (i.e., if the legs or feet swell slightly below the bandage or in the treated area), take some bed rest with the legs elevated with 2 pillows. Do this for 1 hour in the morning, and for one hour in the afternoon. If the swelling does not disappear within 3 days using the above instructions, then notify the doctor immediately for further treatment.

3) If bruises and marks appears on the application area, apply a small amount of the post sclerotherapy product provided by the research team (sodium heparin 0,5% cream), two times daily gently rubbing the cream into the skin. This must be done for two weeks or until the marks and bruises disappear. If the marks/bruising fail to disappear after following these instructions, contact the doctor immediately.

4) If you experience symptoms such as swelling around the lower limb, redness, warmth, or stinging at the application site, contact the staff team.

5) Avoid sun exposure during the first month following the procedure. If impossible, you must use a 30 factor sun block cream/lotion (at least) before you expose yourself to the sunlight.

6) Contact the research staff, at any time, at (14) 3811-6305.

Vascular Surgery Service.

Department of Surgery and Orthopedics, UNESP.

Responsible Doctor: Prof. Matheus Bertanha, M.D., Ph.D.

CRM: 113.496 - Vascular Surgeon.
