# Supplementary material for: Polidocanol versus hypertonic glucose for sclerotherapy treatment of reticular veins of the lower limbs: study protocol for a randomized controlled trial
Source: Trials. 2014 Dec 19;15:497. doi: 10.1186/1745-6215-15-497 (PMC4301449; doi:10.1186/1745-6215-15-497)
Supplement: Supplementary file 5 — Additional file 5: Primary end point. Score of disappearance of reticular veins treated. (DOC 34 KB) [file 13063_2014_2369_MOESM5_ESM.doc]

| **OUTCOMES** | | |
| --- | --- | --- |
| **PRIMARY ENDPOINT – Treatment Efficacy** | | |
| **RESULT** | **DESCRIPTION** | **GRADE** |
| **Excellent** | Complete healing at the target area | **5** |
| **Very good** | Presence of up to 1 cm of non-healed veins in length at the target area | **4** |
| **Good** | Presence of 1 cm to 3 cm of non-healed veins in length at the target area | **3** |
| **Fair** | Presence of 3 cm to 5 cm of non-healed veins in length at the target area | **2** |
| **Bad** | Presence of 5 cm to 9 cm of non-healed veins in length at the target area | **1** |
| **Unsuccessful** | Presence of more than 9 cm of non-healed veins in length at the target area | **0** |
