# Supplementary material for: Polidocanol versus hypertonic glucose for sclerotherapy treatment of reticular veins of the lower limbs: study protocol for a randomized controlled trial
Source: Trials. 2014 Dec 19;15:497. doi: 10.1186/1745-6215-15-497 (PMC4301449; doi:10.1186/1745-6215-15-497)
Supplement: Supplementary file 6 — Additional file 6: Secondary end point. Score of pigmentation. (DOC 32 KB) [file 13063_2014_2369_MOESM6_ESM.doc]

| **OUTCOMES** | | |
| --- | --- | --- |
| **SECUNDARY ENDPOINT – Treatment Safety** | | |
| **RESULT** | **DESCRIPTION** | **GRADE** |
| **Excellent** | No visible hyperpigmentation at the target area | **5** |
| **Very good** | Presence of only one point of hyperpigmentation up to 1 cm in length at the target area | **4** |
| **Good** | Presence of 2 to 3 points of hyperpigmentation up to 1 cm in length, at the target area | **3** |
| **Fair** | Presence of 4 to 5 points of hyperpigmentation up to 1 cm in length, or one hyperchromatic line, up to 5 cm in length, at the target area | **2** |
| **Bad** | Presence of more than 5 points of hyperpigmentation up to 1cm in length, or one hyperchromatic line, with more than 5 cm and less than 9 cm in length, at the target area | **1** |
| **Unsuccessful** | presence of a hyperchromatic line larger than 9cm in length, or the total extension of the vein presenting hyperpigmentation, at the target area | **0** |
